# Evaluation of Adsorptive Characteristics of Cow Dung and Rice Husk Ash for Removal of Aqueous Glyphosate and Aminomethylphoshonic Acid

**DOI:** 10.1038/s41598-019-54079-0

**Published:** 2019-11-27

**Authors:** Jamilu Garba, Wahid Abd Samsuri, Radziah Othman, Muhammad Saiful Ahmad Hamdani

**Affiliations:** 1 Deparment of Soil Science, Faculty of Agriculture, Ahmadu Bello University, 1044 Zaria, Nigeria; 2 Department of Land Management, Faculty of Agriculture, Universiti Putra Malaysia, 43400 UPM Serdang, Selangor Malaysia; 3 Department of Crop Science, Faculty of Agriculture, Universiti Putra Malaysia, 43400 UPM Serdang, Selangor Malaysia

**Keywords:** Environmental sciences, Pollution remediation

## Abstract

Glyphosate (GLY) is a major herbicide used throughout the world, and its continuous application has become an environmental issue. Adsorption is an important mechanism for removing organic contaminant in water. The present study characterized cow dung (CD) and rice husk ash (RHA), and determined the adsorption-desorption of GLY and its metabolite, aminomethylphoshonic acid (AMPA), on to them. The results revealed that both CD and RHA were alkaline and had no or low content of arsenic, cadmium, chromium and lead. The CD had lower surface area (13.104 mg^2^g^−1^) than RHA (21.500 m^2^g^−1^). The CD contained amines, phenol, ethers and carboxylic functional groups, while in addition to carboxylic and ether, RHA contains siloxane. Both CD and RHA had high affinities for GLY and AMPA. The Freundlich sorption coefficient (K_f_) on AMPA were 2.915 and 2.660 for CD and RHA, respectively, while the values on GLY were 1.168 and 1.166 (mg g^−1^) for CD and RHA, respectively. Desorption of GLY only occurred at lower concentrations, while no desorption of AMPA was recorded, indicating their strong adsorption on CD and RHA. Considering their availabilities and affordable prices, both CD and RHA can be recommended as economical adsorbent for the removal of GLY and AMPA.

## Introduction

Glyphosate {N-(phosphonomethlyl) glycine} (GLY) is a broad spectrum herbicide commonly used in Malaysia and it acts by disrupting the shikimic pathway in plants necessary for the synthesis of essential amino acids-phenylalanine, tyrosine and tryptophan^1^- resulting to the death of plant. GLY is a systemic herbicide consequently, it is applied on foliage and later absorbed by the cuticle and translocated to the various parts of the plant via phloem^2,3^. The GLY residues enter the soil either through root exudation, spray drift or due to direct surface application. Increasing application of GLY is of environmental concern because it can pollute soil and water. Soil properties and environmental factors such as rainfall determines the mobility of GLY, thus, it is susceptible to leaching and ground water contamination if applied in soil with low content of oxides minerals and high hydraulic conductivity^4^. Surface- applied GLY can move along with soil particles via runoff due to heavy rainfall or irrigation and emptied into nearby reservoir. The GLY toxicity effects of growth inhibition and mortality was reported on aquatic habitant^5,6^. Similarly, GLY and its degradation product aminomethylphosphonic acid (AMPA) widely occur in surface water, sediment and underground water^7^. For instance, median and maximum GLY concentration of 0.2 and 427 µg L^−1^, respectively was detected from 374 samples of ditches and drains water in USA while, of 116 samples collected from soil water, the median and maximum GLY concentration was <0.02 and 1.00 µg L^−1^, respectively^8^. In Brazil, a concentration range between 1.26 to 1.48 mg L^−1^ of GLY was reported in surface^9^. On one hand Piel *et al*.^10^ reported that, AMPA occurs widely in surface water of France, with higher concentration in urban water (5 µg L^−1^) than in rural water (2 µg L^−1^). Given these instances, removing GLY and AMPA from the water body is of great environmental significance. Methods of waste water treatments such as filtration, chemical coagulation and clarification are only effective at removing pesticides that have low water solubility^11^. Other constraints associated with these methods are high operational and maintenance cost, complicated procedure in their operation and generation of toxic compounds^12^. For these reasons, adsorption serve as an effective method of removing water-soluble pesticide like GLY from water^13^. GLY is zwitterion, containing both negative and positive charges in aqueous phase depending on solution pH. Therefore, its adsorption mechanisms has been suggested to be H-bonding^14^, ligand exchange^15^, surface complexation^16^, precipitation reaction, coordination bonding^17^ and charge transfer^18^.

Activated carbon is the widely used adsorbent for removal of pollutant from water, however, due to its high cost and loss of adsorption efficiency after regeneration led to focusing on the use of low-coast, non-conventional adsorbents as alternative. Different materials have been used in adsorptive removal of GLY, including clay substances^19,20^, humic substances^14^, water and industrial residues^21^, and biochar^18,22–24^. To date there is no reported study on the use of cow dung or rice husk ash for adsorptive removal of GLY and its metabolite, AMPA, from aqueous phase. Cow dung (CD) is the faeces of bull, cow, heifer and calves and is usually added to soil as animal manure due its nutrient contents. Rice husk ash (RHA) is a by-product of rice husk and is burned using cyclonic furnaces serve to as source of fuel for rice driers^25^. It is presumed that these organic materials have higher surface areas and macro and micro pores capable of retaining GLY and AMPA molecules. Likewise, their functional groups and cation contents enhance their adsorption capacities in relation to GLY and AMPA. These agricultural wastes are abundantly available in Malaysia at a very low cost. Therefore, their utilization in adsorptive removal of GLY and AMPA can serve as an alternative method of waste recycling and management. The present study therefore aimed to characterize CD and RHA and investigate their adsorption-desorption for GLY and AMPA in a single-solute system.

## Materials and Methods

### Cow dung and rice husk ash samples

The cow dung was obtained from the animal section of the Experimental Farm (Lat. 2.986460°N, Long. 10.173313°E), Faculty of Agriculture, Universiti Putra Malaysia, while the RHA was obtained from BERNAS rice mill (Lat. 3°40′32.4″N, Long. 100°59′42.5″E), Selangor-Malaysia. The cow dung and rice husk ash were collected from five different locations in their respective sampling areas. The sampling of cow dung and rice husk was carried out after obtaining a permission from the director Universiti Putra agricultural park and the manager BERNAS rice mill, respectively. The cow dung sampling was carried out at the back yard of these livestock fences, therefore did not involve any one of them neither any endangered or protected species. The cow dung was collected from different locations within the backyard, then properly air-dried at room temperature, thoroughly mixed and bulked to one composite sample. The air-dried CD sample was later grounded with pistol and mortar, after which, both CD and RHA were separately passed through a 1 mm sieve and stored in a glass container prior to analysis.

### Chemicals and reagents

Millipore^®^ Direct UV-Q water was used to prepare all the solutions in this study. GLY of 99.7% purity, AMPA of 99% purity and 9-flourenylmethlylchloroformate chloride (FMOC) of 97% purity were purchased from Sigma Aldrich^®^ (Seelze, Germany), while acetonitrile was purchased from QREC^®^, Malaysia. Stock solutions (500 mg L^−1^) of GLY and AMPA were prepared by quantitatively dissolving their appropriate quantities into Millipore^®^ Direct UV-Q water, while 0.02 M FMOC was prepared in acetonitrile. Analytical reagent grade CaCl_2_ and KH_2_PO_4_ were purchased from Emsure^®^ (Germany) while HgCl_2_ and Na_2_B_4_O_7_.10H_2_O were purchased from Sigma Aldrich^®^ (India).

### Cow dung and rice husk ash characterization

The moisture content was determined by oven drying 5-g air dried sample at 105 °C for 24 h, and the moisture content was calculated by the difference between the weight of the fresh and oven-dried samples. Ash content was determined using the method described by Samsuri *et al*.^26^. Briefly, 1 g of either CD or RHA was combusted at 700 °C in muffle furnace for 12 h, and later the ash content was calculated as the difference between the initial and final weights. The pH and EC of the samples were determined using a 0.5:100 (w/v) sample and water suspension. The mixture was shaken on a rotary shaker for 1 hour and then allowed to settled, after which the pH was measured with Matrohm 872 pH mater while the EC was measured with Eutech CON 700 EC meter. The C, H, O, N and S contents in the organic materials were determined using a CHNSO elemental analyser (LECO® Truspec CHNSO). The CEC and extractable bases were measured using the modified method of Song and Guo^27^. Briefly, 0.5 g of CD or RHA were saturated with 40 mL of 1 M NH_4_OAc, shaken overnight and then filtered using a vacuum pump. Immediately, another 40 mL of fresh 1 M NH_4_OAc was added, and the filtrates were combined. After that, 90 mL of ethanol was added to the samples, and the filtrates were discarded. The samples were later leached with 100 mL of 1 M KCl and the leachates were collected to determine the NH_4_ concentration using Lachat Instruments QuickChem 8000 series FIA+ System auto analyser. The CEC was calculated from the concentration of NH_4_^+^ in the 1 M KCl leachate. The concentrations of the extractable bases in the NH_4_OAc filtrates were determined using a Perkin Elmer AAnalyst 4000 atomic absorption spectrometer (AAS).

The total P, Fe, Mn, Zn, Al, Si, Cu, As, Cr, Cd and Pb were determined by digesting 0.25 g of CD or RHA in a digestion vessels. This was achieved through the addition of 7 mL concentrated HNO_3_ and 3 mL of H_2_O_2_ to the samples. Later, the vessels containing the sample mixture were placed in ETHOS1 (Milestone, Italy) microwave digestion system. The vessels were heated for 20 min at 200 °C and 1.2 kW power level. After cooling to room temperature, the digests were filtered into 100 mL volumetric flasks and brought to volume with Millipore^®^ Direct UV-Q water. Total P was determined using a Lachat Instruments QuickChem 8000 series FIA+ System auto analyser while Fe, Mn and Zn were determined using a Perkin Elmer^®^ AAnalyst 4000 AAS. The concentrations of Al, Si, Cu, As, Cr, Cd and Pb in the digest were determined using a Perkin Elmer^®^ Optima 8300 ICP-OES spectrometer. All the analysis were carried out using triplicate samples.

### Determination of surface area and morphology of cow dung and rice husk ash

The surface area of CD or RHA was measured with a Quantachrome version 2.01 (Quantachrome AS1Win^TM^) Autosorb 1 surface area analyser. The samples were degassed at 300 °C then adsorption-desorption with liquid nitrogen was carried out at −195.55 °C, and Brunauer-Emmett-Teller (BET) and Barrett-Joyner-Halenda (BHJ) techniques were used to determine the surface area, pore volume and pore radius of the CD and RHA samples. The surface morphology was determined using scanning electron microscopy (JEOL, JSM-6400V, Japan). The samples were platinum coated by a vacuum electrical sputter coater for 3 min before the measurement was taken.

### Functional groups analysis of cow dung and rice husk ash

The percentages of oxygen-acidic functional groups in the CD or RHA was determined by the Boehm titration method^28^. Briefly, 0.2 g of CD or RHA in triplicate were soaked with 20 mL of either 0.1 M NaOH, 0.1 M Na_2_CO_3_ or 0.05 M NaHCO_3_ solution and shaken for 24 h. The mixture was filtered, and 15 mL 0.1 M of HCl was added to 10 mL of the filtrate then back titrated with 0.1 M NaOH. The absorbance spectra of CD or RHA for surface functional groups was recorded using a Perkin-Elmer^®^ model 1725 Fourier transformed infrared (FTIR) spectrometer (Norwalk, USA) from 280 cm^−1^ to 4500 cm^−1^.

### Batch equilibrium sorption study

This study was performed separately for each of the organic materials, and for contact time up to 24 h at room temperature and at the original pH of GLY and AMPA solutions (4.81–5.3). The study was performed was according to the method of Piccolo *et al*.^14^, with some modifications. In brief, a 0.5 g sample of CD or a 0.3 g of RHA was added separately to a centrifuge tubes containing 20 mL of GLY or AMPA solutions at different concentrations. The initial GLY concentrations were 0, 25, 50, 100, 150, 200, 250, 300 mgL^−1^, while the AMPA concentrations were 0, 4, 8, 17, 25, 33, 42, 50 mgL^−1^. The GLY and AMPA solutions were prepared in 0.01 M CaCl_2,_ with 200 mgL^−1^ HgCl_2_ acting as a bioinhibitor. The centrifuge tubes were shaken for 24 h on a rotary shaker at 100 rpm and then centrifuged at 10,000 rpm for 10 min. The supernatant was decanted and passed through 0.45 µm HmbG syringe filter model P0377 prior to analysis. Desorption study was performed immediately after the supernatants of adsorption study were decanted. In this process, 20 mL solution of 0.01 M CaCl_2_ containing 200 mgL^−1^ HgCl_2_ were added to each centrifuge tube and shaken for 24 hr under the same experimental conditions explained above. The samples were treated similarly to those of the adsorption study prior to analysis.

### Analysis of GLY and AMPA

The analysis of GLY and AMPA in the solution was performed according to a method developed earlier^29^. Briefly, 1 mL of either standard solution or extract was mixed with 1 mL of 0.02 M FMOC-Cl solution in the presence of 2 mL 0.05 M borate buffer. The mixture was derivatized through shaking it on an end-to-end shaker at 180 rpm for 1 h. After that, 2 mL of diethyl ether was added to the mixture and vortex mixed for 2 minutes. Later, the surface organic layer containing excess FMOC was removed. The aqueous solution containing GLY or AMPA was analysed using an Agilent 1100 series of high performance liquid chromatography (Agilent tech. Inc. Germany) equipped with an autosampler injector and a fluorescent detector. The mobile phase solvent was acetonitrile and a 0.05 M KH_2_PO_4_ mixture (30:70 v/v) using isocratic mode and a flow rate of 0.7 mL min^−1^. The column was Agilent^®^ Zorbax Eclipse plus C_18_ (4.6 × 150 mm, 5 µm), and the injection volume used was 20 µl. The detection was made at 270 nm and 315 nm for excitation and emission modes, respectively.

### Data analysis

Adsorption capacity which determine the amount of GLY and AMPA sorbed by CD or RHA was calculated using the equation below:

1 $${q}_{e}=\frac{({c}_{o}-{c}_{e})}{m}V$$

The percent amount of GLY and AMPA removed from the aqueous solution was calculated by the following formula:

2 $${\rm{Removal}}( \% )=\frac{Co-Ce}{Co}\times 100$$

The Freundlich’s and Langmuir’s adsorption isotherm models were applied to the experimental data in order described the adsorption behaviour of GLY and AMPA, and their non-linear equations are shown below:

3 $$\mathrm{Freundlich},\,\,\,{q}_{e}={K}_{f}C{e}^{n}$$

4 $$\mathrm{Langmuir},\,\,\,{q}_{e}=\frac{Qmax\,bCe}{1+bCe}$$

where q_e_ is the amount of compound adsorbed per unit weight of the organic material (mg g^−1^), m is the amount of the adsorbent (g), V represent the solution volume (L), Co is the initial concentration of the compounds in the solution (mg L^−1^), Ce is the equilibrium concentration of the compounds in the solution (mg L^−1^) and K_f_ and n are the Freundlich constants, related to capacity and intensity of adsorption, respectively^15,24^. Q_max_ and b are the Langmuir constants which are the maximum adsorption capacity (mg g^−1^) and a constant related to affinity, respectively. The values of K_f_, n, Q_max_ and b were determined from linearized form of the Freundlich and Langmuir equations^30^.

## Results and Discussion

### Physicochemical properties of cow dung and rice husk ash

The results of the chemical analysis of CD and RHA are presented in Table 1. The pH levels of both the CD and RHA were alkaline, and the EC values were high. The resultant pH (8.14) and EC (2183.33 µs/cm) of the CD can be attributed to the livestock’s management system. They were being kept in a semi-intensive system, which allows them to graze freely and later be supplemented with concentrates that might contain Na, Ca and Mg. This can result in the presence of these salts in the faeces of the livestock, leading to high pH and EC. The resultant values of pH (9.93) and EC (3320 µs/cm) of the RHA were due to its ash content stemming from the cellulose and hemicellulose components of the parent rice husk that were destroyed during the combustion process^25^. The percent ash content in the CD and RHA were 25.33% and 92.33%, respectively, while the respective moisture content of CD and RHA were 41.38% and 1.22%. The results show that the CD contained 30.78% C, 4.79% H, 40.26% O, 2.53% N and 0.23% S, while RHA contained 1.83% C, 1.93% O and 0.19% S, and H and N were below detection levels, which can be attributed to their oxidization during combustion. The calculated H/C and O/C elemental ratio in the CD were 1.87 and 0.98, respectively. Meanwhile, the H/C ratio was not calculated for the RHA because the H element was below detection level of the machine but its calculated O/C ratio was 0.08. Organic materials lost their H, C and O through dehydration and decarboxylation when subjected to heating^31,32^. Therefore, heating of organic materials can resulted into a decrease of its H/C and O/C ratio^18,33^. The resultant values of O/C ratio of the RHA could be attributed to heating and it reflect high content of unsaturated carbon from the RHA. Meanwhile the resultant values of O/C ratio of the CD indicated the presence of water molecules. The P contents of the CD (2.16 gkg^−1^) was higher than that of the RHA (1.73 gkg^−1^), and the same was true of the content of extractable bases. However, the metal cations (As, Cd, Cr, Pb) were below the detection limit in the RHA, whereas Cr and Cd were detected in the CD, which can be attributed to the concentrates being fed to the animals. Cations were reported to form complexes with GLY via its phosphonic moiety in the order of trivalent > divalent > monovalent^34^.

**Table 1:** Physicochemical characteristics of cow dung and rice husk ash.

Parameter (in dry weight)	Cow dung	Rice hush ash
pH	8.14 ± 0.04	9.95 ± 0.02
EC µs/cm	2183.33 ± 67.70	3320.00 ± 115.47
Moisture content (%)	41.38 ± 0.48	1.22 ± 0.21
Ash (%)	25.33 ± 3.36	92.33 ± 1.45
C (%)	30.78 ± 2.73	1.80 ± 0.22
H (%)	4.79 ± 0.12	nd
O (%)	40.26 ± 1.18	1.93 ± 0.01
N (%)	2.53 ± 0.14	nd
S (%)	0.23 ± 0.03	0.19 ± 0.01
H/C	1.87^*^	—
O/C	0.98^*^	0.08^*^
P (g kg^−1^)	2.16 ± 0.05	1.73 ± 0.02
Ca (g kg^−1^)	7.94 ± 1.53	1.16 ± 0.27
K (g kg^−1^)	9.16 ± 1.56	1.93 ± 0.12
Mg (g kg^−1^)	3.74 ± 0.55	1.81 ± 0.21
Na (g kg^−1^)	1.14 ± 0.21	0.33 ± 0.11
CEC (cmol_(+)_ kg ^−1^)	34.50 ± 2.94	10.20 ± 0.73
Cu (mg kg^−1^)	17.47 ± 1.09	7.07 ± 0.27
Fe (mg kg^−1^)	6808 ± 5.84	637.47 ± 7.36
Mn (mg kg^−1^)	158.93 ± 6.62	82.13 ± 3.53
Zn (mg kg^−1^)	88 ± 5.21	36.27 ± 2.92
Al (mg kg^−1^)	7952.80 ± 7.82	235.07 ± 6.31
Si (mg kg^−1^)	482.40 ± 5.42	287.60 ± 2.71
As (mg kg^−1^)	nd	nd
Cr (mg kg^−1^)	4 ± 1.01	nd
Cd (mg kg^−1^)	0.80 ± 0.02	nd
Pb (mg kg^−1^)	nd	nd

nd = not detected, * = ratio, ± SE.

### Surface morphology and surface area of cow dung and rice husk ash

**Figure 1:**
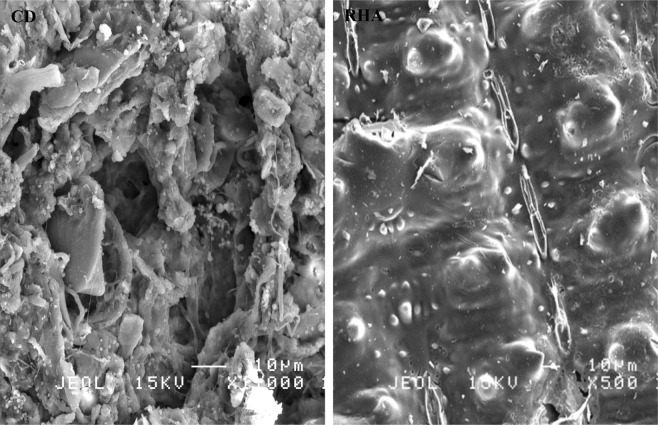
Scanning electron micrographs of (**a**) cow dung and (**b**) rice husk ash.

Figure 1 shows the surface morphology of CD and RHA. The CD surface was rough and porous, with bulky structures filled with rod-like particles, interconnected with thread-like tissues. Meanwhile, the RHA surface was relatively smooth, with globular and porous structures interconnected by feather-like flakes. The porous nature of CD and RHA is an indication of their high surface area owing to the large micro and macro pores, which drive their adsorptive capacity^28^.

**Figure 2:**
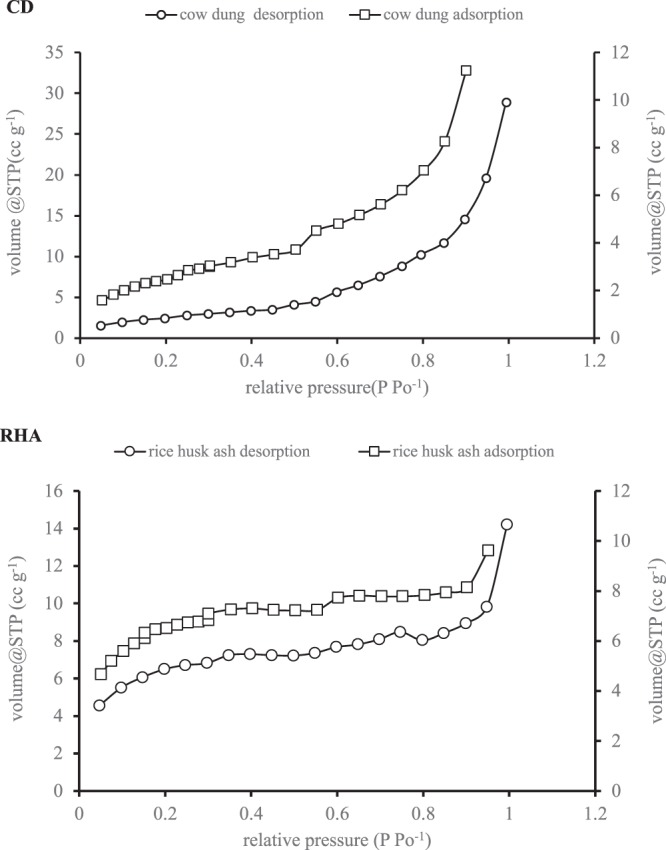
BET adsorption-desorption isotherm of (**a**) cow dung and (**b**) rice husk ash.

Table 2 shows the BET surface area of the CD (9.731 m^2^ g^−1^) and the RHA (21.500 m^2^ g^−1^). The RHA had higher BET surface area but a lower internal surface area (2.743 m^2^ g^−1^) than the CD (13.104 m^2^ g^−1^). The pore volume and pore radius of the CD were 0.046 cm^3^ g^−1^ and 21.451 Å, respectively, and these values are not much different from those of the RHA (0.013 cm^3^ g^−1^ and 24.333 Å). Figure 2 shows the BET adsorption-desorption isotherm of CD and RHA. Both isotherms were linear (C-shape) indicating a constant partitioning between N_2_ and interfacial phase of the CD and RHA surfaces. Thus, adsorption occur without any bonding energy^35^. Surface entrapments is one of the adsorptive mechanism of pesticide removal^36^, which could result to GLY entrapments on the surfaces of CD or RHA.

**Table 2:** Physical characteristics of cow dung and rice hush ash.

Parameter	Cow dung	Rice hush ash
BET surface area (m^2^ g^−1^)	9.731	21.500
Internal surface area (m^2^ g^−1^)	13.104	2.743
pore volume (cm^3^ g^−1^)	0.046	0.013
Pore radius (Å)	21.451	24.333

### Functional groups of cow dung and rice husk ash

The concentration of the total oxygen acidic functional groups present on the surface of CD was 53.60 cmol_(+)_ kg^−1^, with carboxylic, lactonic and phenolic groups at concentrations of 15.12, 19.08 and 19.40 cmol_(+)_ kg^−1^, respectively (Table 3). The total concentration of acidic functional groups on the surface of RHA was 45.58 cmol_(+)_ kg^−1^ (Table 3), comprising carboxylic, lactonic and phenolic groups at concentration of 11.34, 14.84 and 19.40 cmol_(+)_ kg^−1^, respectively. These functional groups are largely determined the sorptive capacity of the adsorbent^27^ because its ionization increases negative charge surfaces for sorption of ionic solute^27,37,38^.

**Table 3:** Oxygen acidic functional groups in cow dung and rice husk ash.

Parameter	Cow dung	Rice husk ash
Total acidity (cmol_(+)_ kg^−1^)	53.60 ± 2. 42	45.58 ± 1.74
Strong acidity (cmol_(+)_ kg^−1^)^a^	15.12 ± 1.64	11.34 ± 0.81
Moderate acidity (cmol_(+)_ kg^−1^)^b^	19.08 ± 0.04	14.84 ± 1.03
Weak acidity (cmol_(+)_ kg^−1^)^c^	19.40 ± 1.04	19.40 ± 0.92

^a^carboxylic acid.

^b^lactones.

^c^phenol.

**Figure 3:**
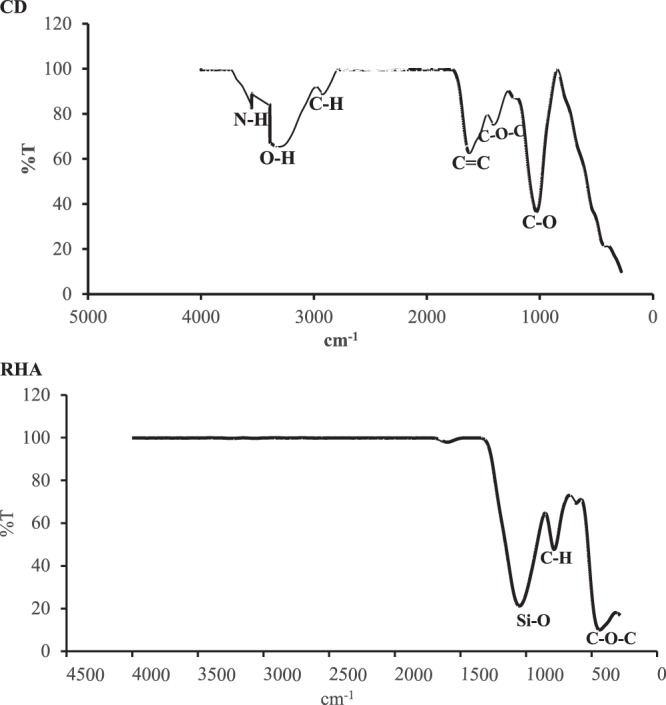
FT-IR spectra of (**a**) cow dung and (**b**) rice husk ash.

Bands from several functional groups were observed on the FT-IR spectra of the CD and RHA (Fig. 3). The presence of more organic constituents in the CD yielded more functional groups than did the RHA, which had already undergone combustion. The bands observed in the CD were secondary amines stretching (3500 cm^−1^), OH bounded groups of phenols and alcohols (3334 cm^−1^), alkanes stretching vibration (2933 cm^−1^), double-bonded carbon alkenes (1628 cm^−1^), CH_3_ (1409 cm^−1^) and primary alcohol stretching (1032 cm^−1^). The bands found on the RHA spectra corresponded to SiO_2_ stretching (1041 cm^−1^), aromatic out of plane bends (783 cm^−1^), and ethers compounds (442 cm^−1^). The spectra also indicated that Si-O is the most significant band in RHA. The CD contained cellulose and hemicellulose polymers, which are rich in OH and alkanes groups. Equally, its lignin content, which is rich in aromatic rings, results in the presence of alkenes spectra^38–40^.

### Batch equilibrium sorption study

Table 4 shows the percent removal of GLY and AMPA through CD or RHA. The study revealed increasing GLY removal by the adsorbents with increasing initial concentration. Meanwhile, there was inconsistent removal of AMPA; lower initial concentrations showed high removal, which decreased at the intermediate concentrations and later increased at higher concentrations. The result generally shows a higher percent removal of GLY by both CD and RHA compared to AMPA, suggesting that both adsorbents had high adsorption capacity of GLY than AMPA. The CD and RHA had higher content of Fe and Al, which might lead to the formation of metal-GLY complexes^41^. The removal of AMPA from the aqueous solution was more in CD than the RHA. This might be due to the presence of a higher number of functional groups and greater internal surface area of the CD, leading to greater penetration of AMPA molecules into inner reactive sites^14^ of the CD compared to the RHA.

**Table 4:** Percent removal of glyphosate and AMPA by cow dung and rice husk ash from the aqueous solutions.

Glyphosate (%)			AMPA (%)		
Initial concentration (mg l^−1^)	Cow dung	Rice husk ash	Initial concentration (mg l^−1^)	Cow dung	Rice husk ash
0	0	0	0	0	0
25	63	60	4	37	22
50	79	77	8	50	49
100	87	87	17	25	20
150	90	90	25	49	34
200	93	93	33	65	51
250	94	93	42	60	59
300	94	95	50	67	62

**Figure 4:**
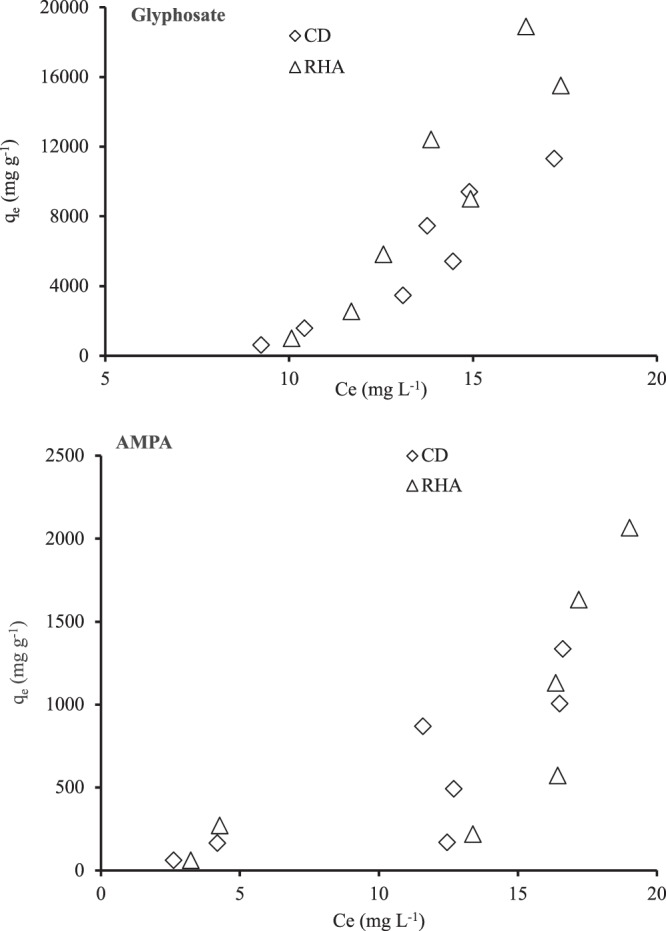
Adsorption isotherms of (**a**) glyphosate and (**b**) AMPA by cow dung and rice husk ash.

The adsorption isotherms of GLY and AMPA for both the CD and the RHA are shown in Fig. 4. Both isotherms were S-type, which indicated that, at low concentrations, the affinity of both GLY and AMPA was low. However, their affinity with CD and RHA increased with increasing initial concentration^35^.

The adsorption isotherms data for both GLY and AMPA on the CD and RHA were fit to the Freundlich and Langmuir adsorption models. The data fit Freundlich’s model (R^2^ > 0.85) well, but it fit Langmuir’s (R^2^ < 0.1) model very poorly (data not shown). The present results agree with the results of Piccolo *et al*.^15^, who reported a better fit of GLY isotherm data to the Freundlich model than to the Langmuir model. The authors studied GLY adsorption on metal-humic acid with different contact times (6–168 h). With a 12-h contact time, their calculated Freundlich and Langmuir R^2^ values were 0.942 and 0.547, respectively. Table 5 shows the values of the adsorption constants for the Freundlich isotherm model for GLY and AMPA adsorption by both the CD and RHA. The K_f_ values of the CD (1.168 mg g^−1^) for GLY was higher than those of the RHA (1.166 mg g^−1^), but CD had lower n values (3.293) than the RHA (3.428). Equally, the K_f_ values of the CD for AMPA (2.915 mg g^−1^) were higher than those of the RHA (2.660 mg g^−1^), while the n values of the CD and RHA for AMPA were 2.119 and 2.151, respectively. Analysis of the CD and RHA revealed higher contents of CEC, Fe, Mn and Al in the CD. Similarly, the CD had a higher internal surface area and pore volume. Equally, the CD had more carboxylic and lactones functional groups than the RHA. The FT-IR spectra of the CD showed the presence of amines, phenols, alcohol and alkanes, while the most significant band of the RHA is Si-O.

**Table 5:** Adsorption constants and correlation coefficients of Freundlich’s isotherm for glyphosate and AMPA adsorption on to the cow dung and rice husk ash.

Freundlich’s sorption parameters				
Adsorbent	Compound	K_f_ (mg g^−1^)	n	R^2^
Cow dung	Glyphosate	1.168	3.293	0.985
	AMPA	2.915	2.119	0.865
Rice husk ash	Glyphosate	1.166	3.428	0.981
	AMPA	2.660	2.151	0.872

These properties of CD could provide its higher adsorption capacity and greater affinity for GLY and AMPA compared to RHA. It was postulated therefore, that more complexes of GLY or AMPA and metals would form in the CD than in the RHA due to the higher content of Fe, Mn and Al in the former. In addition, electrostatic attraction and H-bonding, which are among the mechanisms of GLY and AMPA adsorption, could have taken place between the moieties of GLY and AMPA and functional groups of the CD. The higher internal surface area and pore volume of the CD could have entrapped more GLY or AMPA ions than the RHA. Ucun *et al*.^36^ reported that the bioadsorption mechanisms include ion entrapment in inter- and intra-fibrillary capillaries and spaces of adsorbent constituents. Meanwhile, Piccolo *et al*.^14^ in their study of GLY adsorption on different European soils reported that GLY adsorption with soil humic materials involves a relatively weak bond such as H-bonding. Earlier, Piccolo *et al*.^40^ suggested the formation of complex between GLY and polyvalent cations, especially Fe and Al metals. Therefore, since bioadsorption is not restricted to only one mechanisms, in the present study, the mechanisms of GLY and AMPA adsorption by the CD might be complexation reaction between the compounds and metals of the adsorbent, formation of H-bonding between the phosphonic moieties of the compounds and hydrogen nucleus of the CD’s functional groups, while the mechanisms for GLY and AMPA adsorption by the RHA might be complexation reaction and ions entrapment. The present study shows higher values of K_f_ but lower n values for AMPA adsorption by CD and RHA than for GLY adsorption. This revealed that the CD and RHA had higher affinity to AMPA than to GLY. Since ion entrapment is among the suggested adsorption mechanisms for AMPA and GLY, the lower molecular weight (111.04 g mol^−1^) of AMPA compared to GLY (169.09 g mol^−1^) might be responsible for the former penetrating further into the spaces of CD and RHA. The Freundlich isotherm model explains adsorption occurring on heterogeneous and amorphous surfaces having different adsorption energies. Therefore, these findings are not limited to mono-layer adsorption^42^.

The adsorption of GLY and AMPA therefore occurred on multiple layers of the CD and RHA surfaces. The n constant in the Freundlich model describes the isotherm shape^18^, and in the present study, the n values were >1, which indicates that the amount of GLY or AMPA adsorbed increased with increasing initial concentration. However, the Fig. 4 indicated an S-shape isotherm for both compounds with the both materials. Thus, it can be hypothesized that, the adsorption of GLY and AMPA on these materials is cooperative adsorption and can be occur both in mono and multi layers. By comparison, the K_f_ value of GLY adsorption on activated carbon derived from newspaper waste was 2.54^11^, a value higher than what was obtained in the present study. Similarly, Mayakaduwa *et al*.^18^ studied GLY adsorption on wood biochar and reported higher K_f_ values of 7.272. However, Cederlund *et al*.^22^ conducted a study of GLY adsorption on wood biochar and reported very weak adsorption to the extent that the isotherm data was not fitted to the Freundlich model. The RHA has been shown to be an effective adsorbent of indigo carmine dye and heavy metals^43,44^. Rice husk biochar has also been reported to be a very good adsorbent of Zn, Cu and Pb^26^. Therefore, the present study suggested that CD and RHA have the potentials to be utilized for adsorptive removal of GLY and AMPA.

The results of GLY desorption from CD and RHA are presented in Table 6. In general, desorption decreased with increasing GLY concentration to the extent that no data was obtained between the GLY initial concentration ranges of 150 mg L^−1^ up to 300 mg L^−1^. This indicates strong adsorption for GLY by both CD and RHA especially at high GLY concentrations. The presence of AMPA was not detected at any concentration, indicating very strong adsorption between AMPA and both CD and RHA at all concentrations.

**Table 6:** The percentage of glyphosate desorbed from cow dung and rice husk ash.

Initial concentration (mg l^−1^)	Amount of GLY desorbed (%)	
	Cow dung	Rice husk ash
0	—	—
25	8.468	25.691
50	0.562	1.915
100	0.298	0.003
150	nd	nd
200	nd	0.039
250	nd	nd
300	nd	nd

nd = not detected.

## Conclusions

The present study characterized CD and RHA as potential bioadsorbents for GLY and AMPA. The CD had pH of 8.14 while that of RHA was 9.95 indicating their alkalinity. The CEC of CD (34.50 cmol_(+)_ kg^−1^) was greater than that of RHA (10.20 cmol_(+)_ kg^−1^).These facts coupled with their surface area-CD (13.104 mg^2^ g^−1^), RHA (21.500 mg^2^ g^−1^)-and functional groups could make them suitable bioadsorbents of GLY and AMPA. A batch equilibrium adsorption study revealed increasing GLY and AMPA removal by CD and RHA with increasing initial concentrations of the compounds in aqueous solutions. Adsorption of GLY (K_f_ = 1.168 mg g^−1^) and AMPA (K_f_ = 2.915 mg g^−1^) by CD is higher than by RHA (K_f_ = 1.166 mg g^−1^). Meanwhile, AMPA was adsorbed more than GLY by both bioadsorbents, possibly due to lower molecular weight of the former. Desorption of GLY was only observed at lower initial concentrations, while no desorption of AMPA was observed at any concentration range used in this study. It can be concluded that CD and RHA could be a good bioadsorbents for GLY and AMPA removal considering their physicochemical properties and affordability.
